# Harnessing Proliferation for the Expansion of Stem Cell-Derived Pancreatic Cells: Advantages and Limitations

**DOI:** 10.3389/fendo.2021.636182

**Published:** 2021-02-25

**Authors:** Amanda Oakie, Maria Cristina Nostro

**Affiliations:** ^1^ McEwen Stem Cell Institute, University Health Network, Toronto, ON, Canada; ^2^ Department of Physiology, University of Toronto, Toronto, ON, Canada

**Keywords:** human pluripotent stem cell, beta cell, proliferation, islet, diabetes, in vitro differentiation

## Abstract

Restoring the number of glucose-responsive β-cells in patients living with diabetes is critical for achieving normoglycemia since functional β-cells are lost during the progression of both type 1 and 2 diabetes. Stem cell-derived β-cell replacement therapies offer an unprecedented opportunity to replace the lost β-cell mass, yet differentiation efficiencies and the final yield of insulin-expressing β-like cells are low when using established protocols. Driving cellular proliferation at targeted points during stem cell-derived pancreatic progenitor to β-like cell differentiation can serve as unique means to expand the final cell therapeutic product needed to restore insulin levels. Numerous studies have examined the effects of β-cell replication upon functionality, using primary islets *in vitro* and mouse models *in vivo*, yet studies that focus on proliferation in stem cell-derived pancreatic models are only just emerging in the field. This mini review will discuss the current literature on cell proliferation in pancreatic cells, with a focus on the proliferative state of stem cell-derived pancreatic progenitors and β-like cells during their differentiation and maturation. The benefits of inducing proliferation to increase the final number of β-like cells will be compared against limitations associated with driving replication, such as the blunted capacity of proliferating β-like cells to maintain optimal β-cell function. Potential strategies that may bypass the challenges induced by the up-regulation of cell cycle-associated factors during β-cell differentiation will be proposed.

## Introduction

Major advances in stem cell differentiation protocols for β-cell commitment have taken place throughout the past decade. Early reports that demonstrated the production of insulin^+^ cells using *in vitro* differentiation protocols found that these cells were polyhormonal and did not form monohormonal β-cells *in vivo* (1–3). Subsequent studies have established that tightly-regulated sequential steps in differentiation that mimic human fetal islet development are required and that NKX6-1 expression is essential to generate pancreatic progenitors (PPs) that form monohormonal cells expressing both C-peptide and NKX6-1, which will be referred to as β-like cells in this review (4–9). Stem cell-derived β-like cells demonstrate similar, yet not identical, characteristics to primary adult β-cells, including limited glucose-responsive insulin release, calcium dynamics, and mitochondria-driven oxidative phosphorylation (5, 8, 10–12). A defining feature of primary β-cells is their consistently low proliferation rate throughout adulthood, and the guided differentiation of human pluripotent stem cells (hPSCs) towards monohormonal β-like cells mimics this loss of replication, limiting the final number of cells available for further *in vitro* characterization or *in vivo* transplantation analyses. Promoting cell proliferation *in vitro* to generate higher yields of β-like cells can serve as a strategy to create a sufficient number of β-like cells for the reversal of hyperglycemia in diabetic patients. The following report will review current literature on the proliferation capacity of β-cells from primary human islets and in hPSCs differentiated to PPs and β-like cells. The balance between replication and maturation in stem cell-derived β-like cells and methods to increase total β-like cell yield for their potential application in therapeutics will be discussed.

## Current Evidence of Human β-Cell Proliferation

### Replication During Fetal Development

β-cell proliferation rates during human islet development have been observed in multiple reports, but the limited availability of human fetal pancreatic tissue samples prevents the level of examination established with rodent models. What is evident from the current information available is that fetal pancreatic samples have comparatively high rates of replication in insulin-expressing cells when assessed against adult tissue samples. β-cell replication has been reported to be retained at ~3% from 10–23 weeks of gestational age in fetal pancreata, but is typically reduced to less than 1% in children less than 2 years old, although high variation in the percentage of proliferating β-cells has been noted during the first year of infancy (up to 5.28% KI67^+^ β-cells) (13–18). Early stage (7.5 to 9.5 weeks of gestational age) human fetal pancreata transplanted under the kidney capsule of SCID mice demonstrated increased proliferation in progenitor PDX1 pancreatic cells and in early insulin^+^ endocrine cells, but not in NGN3^+^ endocrine-committed cells, which may suggest that the maturation status of endocrine cells is inversely related with cell replication (19). Further assessment of the correlation between proliferating insulin-expressing cells and their expression of maturation markers is required to conclude this.

### Replication in Mature β-Cells

Low replication is found throughout all endocrine cell types of the adult islet, with approximately 0.38% of islet cells replicating in adults at one time (17). The human β-cell has been reported to have low (less than 0.5%) to no replication occurring under physiological conditions (17, 20). However, a recent report identified subsets of β-cells from donors that are highly proliferative (21). KI67^+^ β-cells expressed distinct cell surface markers (CD9, CD44, CD49F, PDGFRA) and up-regulated signaling through ERK1/2, STAT3, and STAT5, potentially providing novel markers for identifying proliferating β-cells. Differences in β-cell replication rates within the developing and mature islet are likely due to the maturation status of cells. Fetal β-cells demonstrate a functionally immature phenotype when compared to adult cells and favour cell proliferation over glucose metabolism (10, 22, 23). The changes in fetal versus adult β-cell replication may also be driven by differing mechanisms of shared pathways. For example, activation of Glucagon-Like Peptide 1 Receptor (GLP-1R), which classically potentiates insulin release, also drives proliferation in adolescent, but not adult, β-cells through calcineurin -regulated transcription of cell cycle-associated genes (24). Further examination of developing β-cells can therefore reveal unique systems for driving replication.

### β-Cell Proliferation in Diabetes and in Pregnancy

Reports on β-cell replication in patients with diabetes have contrasting findings. Islets from patients with type 1 diabetes do not display increased β-cell proliferation when compared to proliferation in control patient β-cells (16). However, patients with “recent onset” of type 1 diabetes, defined as less than 18 months since diagnosis, demonstrated a 10 times higher rate of β-cell replication compared to control patient β-cells, and this was not observed in patients with long-term type 1 diabetes or in patients with type 2 diabetes (25). This study found that islets from donors with recent onset type 1 diabetes, presenting with insulitis, had higher replication than islets without insulitis, suggesting that inflammation may drive proliferation during this short time frame. β-cells from patients with type 2 diabetes demonstrate unchanged proliferation when compared to control patients, yet proliferation is up-regulated in nondiabetic obese patients (26). This may mark a compensatory mechanism associated with balancing potential obesity stresses present in the period before gradual β-cell failure and apoptosis in type 2 islets. Recent reporting also suggests that human islets undergo adaptive mechanisms under pregnancy to increase the β-cell compartment, and that cadaveric sections from pregnant patients at later gestational ages (32–40 weeks) had increased β-cell proliferation when compared to β-cells from control patients (27). Insulin^+^ staining near duct cells has been found in obese and pregnant patients and suggests a potential target for β-cell neogenesis, but examining proliferation or neogenesis in humans is limited due to their low detection and the limited availability of samples (18, 27–29). However, it does remain that inducing β-cell expansion can serve as a therapeutic for insulin restoration in patients with β-cell dysfunction.

### Comparison of β-Cell Proliferation Between Rodents and Humans

Though rodent models of diabetes are often used to better understand the *in vivo* function of β-cells, evidence in the literature suggests that human β-cells do not demonstrate the same range of proliferation. The application of multiple techniques to induce proliferation in sorted primary β-cells *in vitro*—such as extracellular matrix supplementation and growth factor treatment—revealed that these techniques readily drove rodent β-cell replication but not human replication (30). Screening primary human β-cells for mitogens that had been previously identified to initiate proliferation in rodent β-cells revealed that many factors were unable to initiate significant replication, with the dual-specificity tyrosine phosphorylation regulated kinase 1A (DYRK1A)-inhibiting compound harmine being the only mitogen that induced significantly increased replication with treatment (31). Indeed, harmine is unique in that it has been repeatedly reported in the literature to induce β-cell and other pancreatic cell replication (32–37). Differing effects on β-cell replication through shared signaling mechanisms between rodent and human β-cells may be due to additional cellular factors that inhibit proliferation, such as the high accumulation of p16 in adult human β-cells (38–40). Thus, careful interpretation of factors that induce rodent β-cell expansion should be taken when examining human β-cells.

## Proliferation rates during hPSC differentiation to β-like cells

### Pancreatic and Endocrine Progenitor Replication

Sources of hPSCs, such as embryonic or induced pluripotent stem cells, maintain the unique characteristic of self-renewal prior to lineage-specific differentiation. In order to give rise to the monohormonal β-like cell type that resembles primary β-cells, hPSCs are differentiated to pancreatic progenitor cells that demonstrate co-expression of transcription factors PDX1 and NKX6-1. These cells contain differentiation capacity towards all major pancreatic cell lineages (exocrine, ductal, and endocrine) and bypass the polyhormonal cell fate observed in C-peptide-expressing cells that have no detectable NKX6-1 (41–44). A significant portion of end-stage pancreatic progenitor cells have been found to still contain the replication marker KI67 (~30%–50%) (5, 45, 46). Although KI67 expression is present, pancreatic progenitors demonstrate up-regulation of the cyclin dependent kinase (CDK) inhibitors p21 and p16, suggesting that these cells may be exiting the cell cycle (47). The specification and proliferative rates of pancreatic progenitors can be affected by factors controlling differentiation. YAP, a factor required for Hippo signaling, has been shown to decrease during the differentiation from posterior foregut-like cells (PDX1/NKX6-1^-^) towards PDX1/NKX6-1^+^ pancreatic progenitors (48). However, chemical inhibition of YAP signaling during this developmental stage decreased both the percentage of PDX1/NKX6-1^+^ cells and of KI67^+^ cells, demonstrating that proper commitment towards pancreatic progenitors is also accompanied by proliferation of this compartment (48).

As pancreatic progenitors proceed to differentiate towards more specified endocrine progenitors, characterized by Chromogranin A^+^/NKX6-1^+^ expression, the expression of proliferation markers continues to decrease (47). In the same study that examined the effects of YAP signaling modification on pancreatic progenitor commitment and proliferation, it was found that promoting YAP activity during endocrine progenitor commitment through to β-like cell differentiation led to increased proliferation in cultured cells (48). However, this reduced the final percentage of monohormonal β-like cells, while inhibiting YAP during these stages increased the percentage of monohormonal β-like cells. The authors of this study tested the CDK inhibitor roscovitine during endocrine progenitor differentiation and found that endocrine progenitor commitment was not affected by CDK inhibition (48). In contrast, disrupting the cell cycle using the compound aphidicolin throughout endocrine progenitor and β-like cell commitment, which arrested endocrine progenitor cells at G1 and inhibited the completion of S phase, improved the differentiation of endocrine progenitor cells to β-like cells, but this effect was not seen in the same degree with CDK inhibition alone (49). This may suggest that compounds that disrupt cell cycle progression in endocrine progenitors enhance differentiation to end-stage β-like cells.

### β-Like Cell Replication

At the stage where monohormonal hPSC-derived β-like cells emerge, only a small subset of end-stage β-like cells are actively replicating (~1%), as demonstrated using common markers of proliferation such as KI67 or 5-ethynyl-2’-deoxyuridine (EdU) incorporation assays (5, 32, 48, 50, 51). As mentioned in the preceding section, modifications to the proliferation of pancreatic or endocrine progenitors can impact the final population of β-like cells, which demonstrates a balance that must be maintained between successful β-like cell production and progenitor cell expansion. Although low proliferation rates in β-like cells follows the events seen during primary β-cell development and maturation, it limits the expansion of hPSC-derived β-like cells for further *in vitro* analyses and *in vivo* transplantation studies. For this reason, the search for methods to target β-like cell-specific replication *in vitro* is under continuous investigation. A notable report from the Melton group demonstrated that the leukemia inhibitory factor (LIF) drove proliferation in β-like cells expressing the corresponding receptor (LIFR). Activation of this pathway induced the expansion of β-like cells *in vitro* and was also able to enhance harmine-induced proliferation in treated β-like cells (32). To improve specificity and minimize the off-target effects from small molecule treatments, a recent study developed a zinc-binding prodrug for the deployment of compounds selectively in zinc-rich β-like cells. The study utilized β-like cells in an *in vitro* 3D platform to screen for molecules that targeted β-like cell replication. This novel platform allowed for the development of a harmine-carrying zinc-binding prodrug that was more effective than harmine alone at expanding β-like cells (37). Therefore, further development of compounds that induce targeted proliferation provide promising therapeutic avenues for both inducing and regulating β-like cell replication.

## Caveats linked to Promoting Proliferation during hPSC Differentiation to β-like Cells

Inducible control over replication during the differentiation of hPSC-derived β-like cells is a potent strategy for restoring the number of β-cells for patients that have insufficient β-cell mass. Before this can be pursued for basic research and potential clinical use, all challenges associated with cell cycle manipulation must be addressed.

A general issue that arises with the use of stem cell-derived cell sources is ensuring that proliferative capacity within end-stage β-like cells is tightly regulated to avoid uncontrolled cell growth. While cellular outgrowths have been occasionally detected following transplantation of cultures containing pancreatic progenitors (3, 7), endocrine progenitors (10), or end-stage cultures that failed to commit to the endocrine lineages (52), no outgrowths have been reported from transplantation of end-stage populations sorted for insulin-expressing cells (10). These findings from insulin-purified cell sorts are not surprising as human embryonic stem cell (hESC)-derived endocrine cells are typically post-mitotic, and there has been evidence that expanded pancreatic progenitors do not induce outgrowth when transplanted to mice (44). However, if proliferation is induced within β-like cells during *in vitro* differentiation, blocking proliferation prior to transplantation would become necessary to eliminate the risk of outgrowths.

The induction of proliferation during hPSC pancreatic differentiation to expand a transplantable population of β-like cells is challenged by reports that have documented inhibited commitment and maturation of pancreatic progenitors to end-stage β-like cells. Attempts made at expanding the pancreatic progenitor population have found that PDX1 cells generated do not necessarily express NKX6-1 and that pancreatic marker expression varied with passage (44, 46). The inverse relationship between proliferation and functional maturation of β-like cells was further supported in a recent study examining the cell surface CD9 marker (52). Depleting the stem cell-derived population of CD9^+^ β-like cells removed the population of immature β-like cells, leaving cells that demonstrated higher expression of genes associated with β-cell maturity and insulin secretion. This study also established that human fetal pancreatic C-peptide^+^/KI67^+^ cells expressed higher frequencies of CD9^+^ compared to the C-peptide^+^/KI67^-^ cells, confirming the higher proliferative status of the CD9^+^ fraction *in vivo*. As mentioned previously, recent findings from the Melton lab identified YAP as one factor that could drive proliferation of β-like cells while reducing their maturation when up-regulated during their differentiation (32, 48). This group had also identified that WNT signaling was found in replicating epithelial progenitor cells and is down-regulated in mature endocrine-like cells (53). The TGF-β receptor inhibitor SB431542 has been shown to induce replication in adult human β-cells (54–56). However, SMAD2/3 inhibition through TGF-β can be detrimental in inducing monohormonal β-like cells when applied during pancreatic progenitor differentiation, instead leading towards early lineage polyhormonal endocrine cells that give rise to glucagon-expressing cells (1, 42). Prolonged TGF-β inhibition through ALK5 can also reduce β-like cell glucose-responsiveness when extended beyond endocrine progenitor commitment during β-like cell differentiation (12). Thus, exit from the cell cycle appears to enhance stem cell-derived cultures towards mature β-like cells *in vitro*. The inverse relationship between the maturation and proliferation of β-like cells can be further supported when looking at studies from primary β-cells in human and rodent islets. Signaling pathways such as NOTCH and mTOR have all demonstrated up-regulation in proliferating β-cells at the cost of their functionality (57–59). These findings present a challenge for successfully promoting proliferation in mature β-like cells since there are valid concerns with proliferation inducing characteristics of immature β-like cells and promoting unregulated expansion of transplanted grafts.

## Potential Strategies and Future Directions

The end goal of stem cell-derived β-like cell research is to develop a transplantable system of cells that fully replicate β-cell function. Ideal tactics to achieve this would be to optimize current differentiation protocols, such as through manual purification of mature populations or treatments that increase final β-like cells (10, 12, 41, 52, 60, 61), and to improve *in vivo* site support for transplanted β-like cells (62–64). Despite the limitations listed above, expanding β-like cells or their progenitors *in vitro* still presents a promising strategy to further support these approaches. The final yield of end-stage β-like cells generated using current hPSC-based differentiation protocols is relatively poor and time-consuming, taking 23 days to 1 month to reach β-like cells (5, 10, 12, 53, 60, 65). Therefore, identifying strategies that promote controlled pancreas-specified cell proliferation *in vitro* could facilitate the manufacturing processes necessary to move forward with translational options.

**Figure 1:**
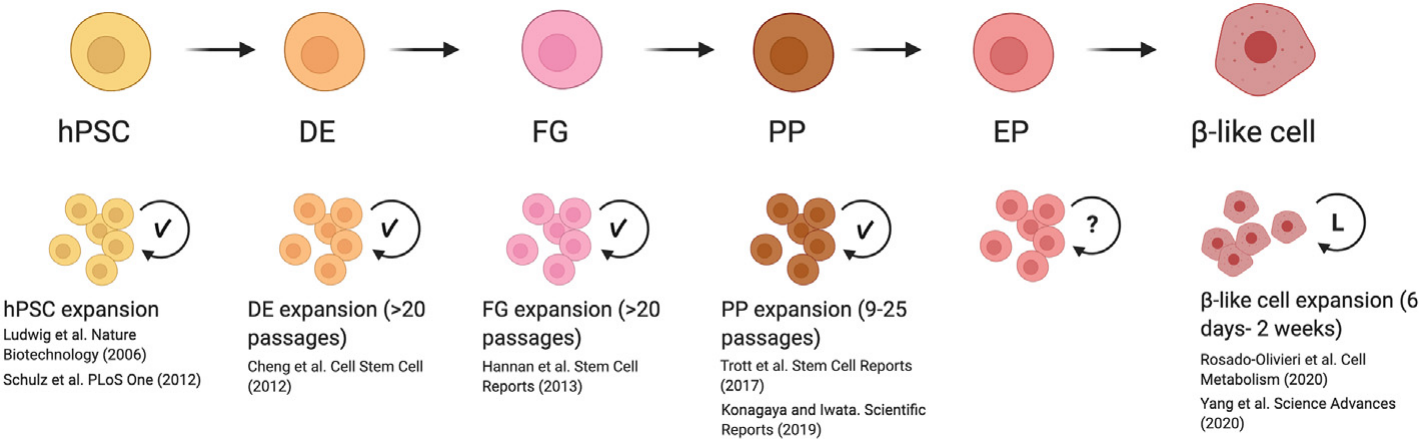
Reported mechanisms and caveats for expanding cells during β-like cell differentiation. Previously reported methods for inducing replication of human pluripotent stem cells (hPSC) to end-stage β-like cells demonstrated that hPSC, definitive endoderm (DE), foregut (FG), and pancreatic progenitor (PP) stages are able to undergo significant expansion with the development of optimized culture conditions (indicated with check mark). These expanded cells are able to maintain their developmental potential with serial passaging. Although proliferation rates have been reported for endocrine progenitors (EP), methods to induce their replication *in vitro* are not well-studied (indicated with question mark). Inducing replication in β-like cells has been successfully initiated. However, β-like cells were only examined after a limited period of expansion *in vitro* (indicated by L), and the long-term effects of driving proliferation are not known. This figure was created using BioRender.com.

Previous studies have identified methods to expand definitive endoderm, foregut endoderm, and pancreatic progenitor populations (**Figure 1**) (44, 46, 66, 67). While these approaches demonstrated the feasibility to induce self-renewal without impacting the developmental potential of hPSC-derived cells, these methods proved difficult to scale up. As a result of this, the generation of pancreatic cells for clinical purposes focuses on the expansion of undifferentiated hESCs, which can be grown and expanded in large bioreactors in the absence of special matrices or stromal cells (8, 68, 69). However, this approach is far from perfect, as a combination of proliferative and apoptotic events occurring during the differentiation process lead to a net outcome of 1 β-like cell for every 2 hESCs seeded (5). Therefore, being able to prevent these losses or expand cells at specific developmental stages will be key to increasing the β-cell yield. Strategies such as throughput screening, previously used to establish proliferative factors in primary β-cells, can be adapted for hESC-derived endocrine progenitor and β-like cell screening (31, 35, 70). With this tool, it is possible to screen for chemical or genetic regulators that provide control over proliferation in pancreatic cells. In addition to using compound-based treatments previously mentioned, such as harmine (34, 35, 37), another method for inducing replication could be through viral delivery of controllable systems (70–72). Once proliferative drivers have been identified, rigorous examination of the expanded population must confirm that no aberrant mutations have been acquired during the process and that expanded cells retain the ability to form β-like cells. Importantly, if proliferation is induced in β-like cells, one would need to show that cell cycle progression can be blocked and confirm that β-like cells retain maturation features, such as the ability to recapitulate first and second phases of insulin secretion in response to glucose challenge and other secretagogues, and that these cells express molecular markers indicative of bona fide β-cells (10, 12, 60).

Ultimately, the successful establishment of controlled proliferation of hESC-derived differentiating pancreatic cells will allow for the large-scale production of glucose-regulating cells and reduce the high costs associated with growing large batches of undifferentiated hPSCs from the initial steps of differentiation. Strict guidelines for the proposed protocols must ensure that the end product population generate islet-like cells with the ability to normalize glycemia without the risk of teratoma or outgrowth formation. The combination of transient expansion with other strategies to optimize β-like cell differentiation will inform the development of an accessible cellular bank for patients, and ideally contribute to their permanent halt of exogenous therapies for glucose management.

## Author Contributions

AO and MCN wrote the manuscript. All authors contributed to the article and approved the submitted version.

## Funding

This work was supported by funding from the Toronto General and Western Hospital Foundation, by a Canada First Research Excellence Fund (CFREF), Medicine by Design and from the Canadian Institute of Health Research project grant to MCN. AO is supported by a Banting and Best Diabetes Centre Postdoctoral Fellowship.

## Conflict of Interest

The authors declare that the research was conducted in the absence of any commercial or financial relationships that could be construed as a potential conflict of interest.
